# Altered Phenotypes in *Saccharomyces cerevisiae* by Heterologous Expression of Basidiomycete *Moniliophthora perniciosa SOD2* Gene

**DOI:** 10.3390/ijms160612324

**Published:** 2015-06-01

**Authors:** Sônia C. Melo, Regineide X. Santos, Ana C. Melgaço, Alanna C. F. Pereira, Cristina Pungartnik, Martin Brendel

**Affiliations:** 1 Departamento de Ciências Biológicas, Laboratório de Biologia de Fungos, Centro de Biotecnologia e Genética, Universidade Estadual de Santa Cruz (UESC), Rodovia Jorge Amado, km 16, Ilhéus, Bahia CEP 45662-900, Brazil; E-Mails: scmelo@uesc.br (S.C.M.); anaclarame@hotmail.com (A.C.M.); bellefp@gmail.com (A.C.F.P.); martinbrendel@yahoo.com.br (M.B.); 2 Departamento de Ciências Naturais, Universidade Estadual do Sudoeste da Bahia (UESB), Estrada do Bem Querer, km 4, Vitória da Conquista, Bahia CEP 45083-900, Brazil; E-Mail: sxneide@gmail.com

**Keywords:** reactive oxygen species, manganese superoxide dismutase, functional heterologous expression, diethylnitrosamine super-sensitivity

## Abstract

Heterologous expression of a putative manganese superoxide dismutase gene (*SOD2*) of the basidiomycete *Moniliophthora perniciosa* complemented the phenotypes of a *Saccharomyces cerevisiae* *sod2Δ* mutant. Sequence analysis of the cloned *M. perniciosa* cDNA revealed an open reading frame (ORF) coding for a 176 amino acid polypeptide with the typical metal-binding motifs of a *SOD2* gene, named Mp*SOD2*. Phylogenetic comparison with known manganese superoxide dismutases (MnSODs) located the protein of *M. perniciosa* (MpSod2p) in a clade with the basidiomycete fungi *Coprinopsis cinerea* and *Laccaria bicolor.* Haploid wild-type yeast transformants containing a single copy of Mp*SOD2* showed increased resistance phenotypes against oxidative stress-inducing hydrogen peroxide and paraquat, but had unaltered phenotype against ultraviolet–C (UVC) radiation. The same transformants exhibited high sensitivity against treatment with the pro-mutagen diethylnitrosamine (DEN) that requires oxidation to become an active mutagen/carcinogen. Absence of Mp*SOD2* in the yeast *sod2Δ* mutant led to DEN hyper-resistance while introduction of a single copy of this gene restored the yeast wild-type phenotype. The haploid yeast wild-type transformant containing two *SOD2* gene copies, one from *M. perniciosa* and one from its own, exhibited DEN super-sensitivity. This transformant also showed enhanced growth at 37 °C on the non-fermentable carbon source lactate, indicating functional expression of MpSod2p. The pro-mutagen dihydroethidium (DHE)-based fluorescence assay monitored basal level of yeast cell oxidative stress. Compared to the wild type, the yeast *sod2Δ* mutant had a much higher level of intrinsic oxidative stress, which was reduced to wild type (WT) level by introduction of one copy of the Mp*SOD2* gene. Taken together our data indicates functional expression of MpSod2 protein in the yeast *S. cerevisiae*.

## 1. Introduction

Aerobic organisms use oxygen (O_2_) as the final electron acceptor in their carbohydrate metabolism. During respiration, some of the O_2_ is only partially reduced, forming reactive oxygen species (ROS) such as anion superoxide (O_2_·^−^), hydrogen peroxide (H_2_O_2_) and hydroxyl radical (OH·). Mitochondria convert 1%–2% of the oxygen consumed into O_2_·^−^ [1]. As these ROS can cause significant cellular stress and damage, antioxidant protection is essential for survival in an aerobic environment. The balance between ROS production and cellular anti-ROS defenses, therefore, determines the degree of oxidative stress [2], which results from the imbalance between oxidants and anti-oxidants in favor of the former [3]. Apart from the endogenous production of ROS during respiration in aerobic organisms, oxidative stress may also be induced by a wide range of environmental factors including UV-light, pathogen invasion, herbicide action or shortage of O_2_ [4].

Cells protect themselves against oxidative damage by different defense mechanisms. These include enzymes (such as peroxidases, catalases and superoxide dismutases (SOD)), anti-oxidants, such as glutathione, vitamins A, C and E [5]; and non-protein complexes of manganese as a back up of ROS-scavenging systems for handling O_2_·^−^ and related ROS [6]. The first defense against O_2_ toxicity involves at least one form of the SOD enzymes [7,8] that promote the conversion of two molecules of O_2_·^−^ into O_2_ and H_2_O_2_; the latter in turn is further degraded by catalase into O_2_ and H_2_O [9].

SOD enzymes (EC1.15.1.1) are characterized in many different organisms and may employ different co-factors to carry out the dismutation of O_2_·^−^, like copper (CuSOD), manganese (MnSOD), iron (FeSOD) or nickel (NiSOD) or combinations like copper and zinc (Cu/ZnSOD) [10]. The Sod1p (Cu/ZnSOD) and Sod2p (MnSOD) are important to improve survival, but so far are only well characterized in yeasts and mammals [11,12].

A few SOD genes from fungi have been characterized. In *Neurospora crassa sod*-1 encodes a major Cu/ZnSOD, and mutant strains lacking this enzyme were sensitive to paraquat and elevated O_2_ concentrations, and exhibited an increased spontaneous mutation rate [13]; evidence was provided for autoreactivity to the human MnSOD in allergic persons sensitized to an environmental allergen from *Aspergillus fumigatus*, which shares a high degree of sequence homology to the corresponding human enzyme [14]; *Colletotrichum graminicola* predicted MnSOD protein did not appear to contain a signal peptide that would target it to the mitochondria, and the expression was associated with differentiation of both oval and falcate conidia [15]; a homologue of yeast and human *SOD1*, MoSod1 from *Magnaporthe oryzae* was identified as Cu/ZnSOD and regulated by MoSir2 to alleviate MoSOD1 transcript repression and detoxify host ROS [16]. In *S. cerevisiae*, the *SOD1* gene (encoding a Cu/ZnSOD protein) is predominantly located in the cytosol, while the *SOD2* gene encodes MnSOD, which is located in the mitochondria. Although located in different cellular compartments, both proteins share the same main role in ROS protection by dismutating O_2_·^−^ into O_2_ and H_2_O_2_ [17,18,19]. In yeast and many other eukaryotes Sod2p (MnSOD) is synthesized by 80S ribosomes and imported into the mitochondrial matrix [20] where it plays an essential role in oxidative stress protection. Apart from its role in dismutation of O_2_·^−^ MnSOD, especially at high activity, may cause cell growth inhibition due to increased production of H_2_O_2_ [21]. Elevated MnSOD activity may change the mitochondrial redox state and thus influence coordination of physiological and biochemical events in cellular compartments [22]. *Beauveria bassiana*, a filamentous entomopathogen, has five distinct SODs, which were proved to contribute to intracellular SOD activity and additively acted in antioxidation and virulence. Subcellular localization of mitochondrial FeSOD (Sod4) and cell wall-anchored Cu/ZnSOD (Sod5) were characterized in this fungus [23].

SODs were found to be an important virulence factor in nearly all pathogenic fungi [24]. *Candida albicans* Cu/ZnSOD is required for the protection against oxidative stresses and for expression of full virulence in human cells. Upon encountering superoxide stress, such as generation of nicotinamide adenine dinucleotide phosphate-oxidase (NADPH oxidase)-mediated O_2_·^−^ species, predominant antioxidant proteins named SOD4 and SOD5 rapidly break down O_2_·^−^ on cell surfaces [25]. Also, SOD influences the virulence of *Cryptococcus neoformans* by affecting its growth within macrophages [26]. However, *A. fumigatus* SODs are intracellular and do not neutralize extracellular ROS in spite of the high sensitivity of this fungus to intracellular ROS generators, thus suggesting that in this case SODs are not putative fungal virulence factors [27].

Apart from modulating virulence of pathogenic fungi, SOD also may have other functions. In the case of *Candida glabrata* the absence of both SODs leads to auxotrophy for lysine, a high rate of spontaneous mutation and reduced chronological lifespan. In a more general context SODs also play an important role in metabolism, acting in biosynthesis, DNA protection and aging [28]. In the yeast *S. cerevisiae*, SOD enzymes play a substantial role in preserving the genomic integrity and their absence leads to shorter life span by allowing increased DNA fragmentation [29]. Also, by triggering production of organic acids, *SOD2* has the potential to promote cell population growth under nutrient deprivation stress [30]. In the yeast *Schizosaccharomyces pombe*, Sod2p is the major salt tolerance plasma membrane protein as it functions in removing excess intracellular sodium (or lithium) in exchange for protons [31].

After successful invasion of the host, phytopathogens, such as *Moniliophthora perniciosa*, can elicit *in planta* either a localized response that is often associated with an oxidative burst or a more generalized systemic response mediated by signaling molecules, or a combination of both [32]. The oxidative burst generates ROS, *i.e.*, O_2_·^−^, OH· and H_2_O_2_, that form a toxic barrier to pathogen invasion [33]. In order to survive the produced ROS, parasites and phytopathogens rely on their anti-ROS defenses for survival under these conditions [34,35,36].

Witches’ broom disease (WBD) of cacao (*Theobroma cacao*), caused by the hemibiotrophic basidiomycete *M. perniciosa*, exhibits a succession of symptoms that are caused during the monokaryotic, biotrophic phase of the fungus that precedes the dikaryotic, necrotrophic phase in *T. cacao.* WBD begins when wind-borne monokaryotic basidiospores infect young meristematic tissues through stomatal openings and form intercellular monokaryotic hyphae, which cause hypertrophy and hyperplasia of the tissues, loss of apical dominance and proliferation of auxiliary shoots, known as “green brooms”. After 3–6 weeks of infection, the homothallic fungus undergoes sexual differentiation, produces clamp connections and forms a dikaryotic mycelium; this marks the transformation from biotrophic to necrotrophic growth phase characterized by the change from inter- to intra-cellular growth; this in turn causes necrosis and death of infected tissues, known as “dry brooms” [37,38,39,40]. Infected tissues (green brooms) present high levels of glycerol and increased accumulation of H_2_O_2_ [40].

The mycelium of *M. perniciosa* in necrotrophic growth phase, either *in vivo* or *in vitro*, is mainly a dikaryon with two nuclei per cell [41,42]. Expression of Mp*SOD2* (*M. perniciosa* homologue of fungal *SOD2* genes) has been monitored in *M. perniciosa* dikaryotic cells. MpSod2p was shown to have constant basal expression when grown either in glycerol or glucose, and was induced after H_2_O_2_ exposure in glycerol grown cells [35,43]. Therefore, this gene is supposed to be an important antioxidant defense of this fungus [35,43] against intense oxidative stress generated by the invaded plant host [39,44,45].

Since Sod2p seems essential in protecting against mitochondria-induced oxidative stress we have transferred its encoding gene Mp*SOD2* into the *S. cerevisiae* mutant *sod2Δ* (yeast mutant allele in which most of the open reading frame (ORF) has been deleted) in order to verify its role in oxidative stress protection via heterologous expression.

## 2. Results and Discussion

In this study we identified via sequence homology and functional heterologous expression the *M. perniciosa* Sod2p-encoding gene. To date, apart from *Saccharomyces and Candida* [46], four other MnSODs from filamentous fungi have been characterized which are from *Aspergillus* [14], *Penicillum* [47], *Ganoderma* [48], *Colletotrichum* [15]. But to our knowledge this is the only one indicating that its heterologous expression in *S. cerevisiae* enhances resistance to ROS and activates the pro-mutagen diethylnitrosamine (DEN).

### 2.1. Characterization of M. perniciosa Manganese Superoxide Dismutase (MpSOD2) Gene and Its Predicted Product

**Figure 1:**
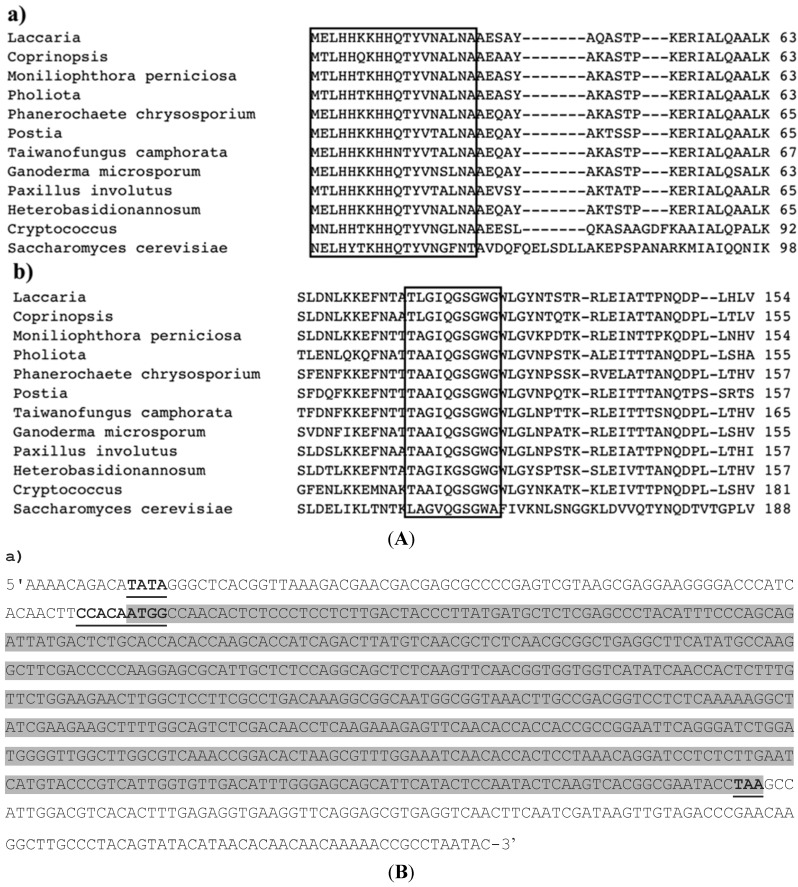

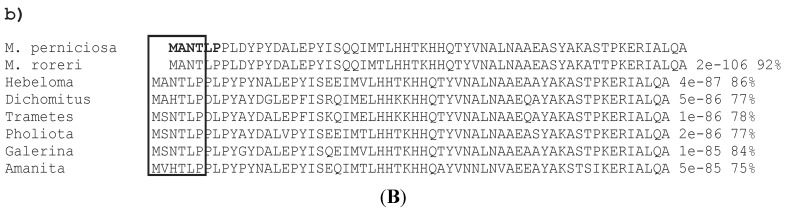
(**A**) Alignment of the Mp*SOD2* deduced amino acid sequence with MnSOD2 polypeptides from other organisms; Conserved residues are boxed in black frame. Fe/MnSod2p (**a**) *N*-terminal α hairpin domain; (**b**) *C*-terminal domain. Protein domains are in a black frame, full alignment not shown; (**B**) Nucleotide sequence of gene Mp*SOD2* and its protein sequence. (**a**) Highlighted are upstream a region defined as sequence of thymine-adenine-thymine-adenine TATA box (marked in bold letters), Kozak motif with start and stop codon (marked in bold letters). The highlighted gray sequence is the CDS (codon DNA sequence); (**b**) Highlighted are upstream region with the following sequence of aminoacids MANTLP (methionine, adenine, asparagine, threonine, leucine, proline) in basidiomycete fungi marked in bold letters from *M. perniciosa* and in a box frame from other organism.

Sequence analysis of a putative Mp*SOD2* clone revealed that it contained a 748 bp insert with an ORF encoding a predicted polypeptide of 176 amino acids (aa) (Genbank accession No. XM2395448). Searches of GenBank using the BLASTP algorithm indicated that the predicted protein was similar to several proteins within the family of manganese-type superoxide dismutases (MnSODs, Figure 1A). Accordingly, the *M. perniciosa* gene was named Mp*SOD2*. A Kozak consensus sequence [49] surrounds the putative *SOD2* start codon (Figure 1B). There is an upstream consensus TATA box motif, but a 3ʹ AT-rich region (a region rich in residues of adenine and timine) is missing. The Mp*SOD2* cDNA contains a 132 bp 3ʹ-untranslated region. A consensus polyadenylation signal (AATAAA; [15]) is absent.

The predicted Sod2p has four conserved aa residues, His-31, His-32, Asp-149, and Pro-155 that are known to coordinate an epitope for metal ligand binding in the MnSOD gene family [50,51,52]. The presence of Gly-69 and Gly-70 instead of Ala-69 and Gln-70 and the absence of a conserved Trp-71 that occurs in proteins of the FeSOD family [53,54] confirm that MpSod2p is affiliated with the MnSOD rather than the FeSOD family. In eukaryotes MnSOD is usually targeted to mitochondria and contains a typical signal peptide rich in basic and hydrophilic aa [55]. The Mp*SOD2* sequence lacks this information and is thus similar to Sod2p of *A. fumigatus* [14], *Penicillium chrysogenum* [46] and *Ganoderma microsporum* [47]. The *N*-terminal aa sequence of MpSod2p was determined and compared with those of Sod2p from other organisms that have the typical 6 aa sequence of M-A-N-T-L-P (Figure 1B). This sequence is part of a conserved domain of iron/manganese superoxide dismutase and, therefore, this specific sequence could be used to identify the *SOD2* gene in basidiomycetes.

**Figure 2:**
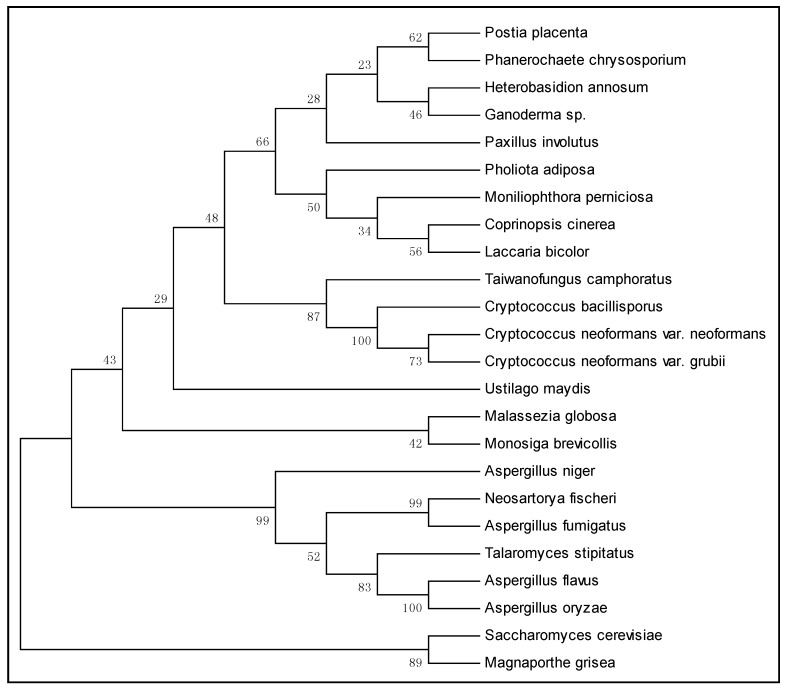
Neighbor-joining tree based on *SOD2* gene DNA sequences of 24 species. Bootstrap values (1000 replicates) are shown under the branches.

Cluster analysis is represented graphically and consists of nodes and branches that summarize evolutionary relationships among particular taxa [56]. By using the neighbor-joining method [57] we could construct a dendogram based on *SOD2* gene DNA sequences of 24 species. That placed *M. perniciosa* close to the basidiomycota *Coprinopsis cinerea* and *Laccaria bicolor* (Figure 2) and, due to low similarity, far from species of ascomycota (*A. fumigatus*, *M. grisea*) and deuteromycota (*A. niger*). *M. perniciosa*, *C. cinerea* and *L. bicolor* form a clade with limited statistical support of 34% and is neighbor clade of the filamentous fungus *Pholiota adiposa* (statistical support of 50%).

According to Blastx (NCBI), gene Mp*SOD2* showed similarity with *P. adiposa* (6 × 10^−17^), with *C. cinerea* (1 × 10^−19^) and *L. bicolor* (1 × 10^−17^). Fourteen species of basidiomycetes formed a clade that was separated from the ascomycetes *A. fumigates* and *S. cerevisiae*, with limited statistical support of 43%.

### 2.2. Functional Expression of MpSOD2 in S. cerevisiae sod2Δ Mutant

**Figure 3:**
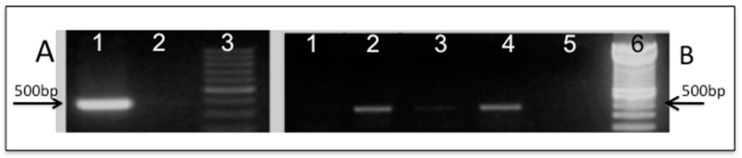
Molecular confirmation of plasmid constructions and yeast transformation by PCR: (**A**) Plasmid pLBF01 containing (1) Mp*SOD2*; (2) pRS313; (3) molecular DNA marker (100 bp ladder); (**B**) Confirmation of yeast transformants containing either pRS313 or pLBF01 (PCR of DNA from each yeast clone): (1) SM01 (WT/pRS313); (2) SM02 (WT/pLBF01); (3) SM03 (Sc*sod2Δ/*pRS313); (4) SM04 (Sc*sod2Δ/*pLBF01); (5) no DNA added to reaction; (6) molecular DNA marker (100 bp ladder).

The putative Mp*SOD2* ORF was shown to be correctly expressed and the homology function confirmed by phenotypic complementation of the yeast *sod2Δ* mutant. Construction of the plasmid named pLBF01 containing putative the putative Mp*SOD2* gene followed by transformation of yeast mutant (Sc*sod2Δ*) and its isogenic WT with both empty (pRS313) and Mp*SOD2*-containing (pLBF01) plasmids (Figure 3A,B) yielded transformants named SM01, SM02, SM03 and SM04, respectively.

Heterologous expression of genes in yeast has the advantage that the probably genetically best-studied eukaryotic organism is used for this purpose [58]. Two genes of *Zygosaccharomyces rouxii* (Z-*SOD22* and Z-*SOD2*) were functionally expressed in a *S. cerevisiae* salt-sensitive mutant and the latter was shown to contribute to the halotolerance in *Z. rouxii* [59]. In addition, a *S. cerevisiae sod1* mutant deficient in Cu/ZnSOD could be complemented by a MnSOD gene from *Bacillus stearothermophilus* [60].

**Figure 4:**
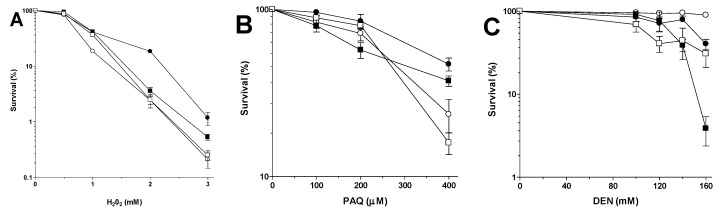

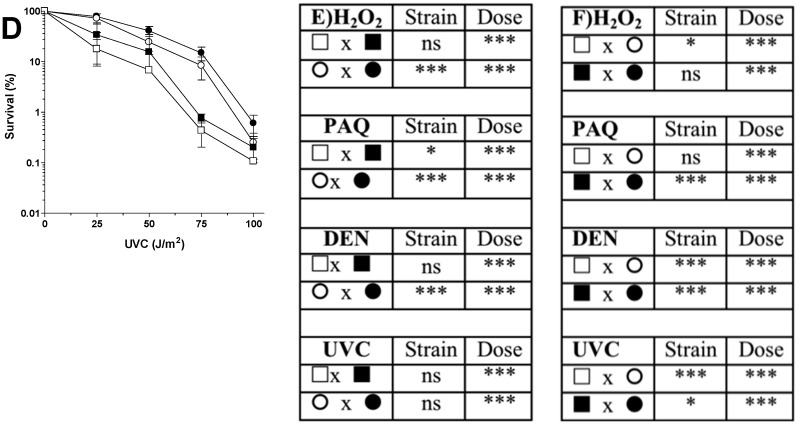
Sensitivity of stationary phase (STAT) cultures of yeast transformants after exposure to (**A**) H_2_O_2_; (**B**) Paraquat (PAQ); (**C**) DEN; and (**D**) UVC. Symbol legend: □ = WT(pRS313); ■ = WT(pLBF01); ○ = Sc*sod2Δ*(pRS313); ● = Sc*sod2Δ*(pLBF01). Statistical analyses of the influence of Mp*SOD2* on the sensitivity phenotype of (**E**) WT and Sc*sod2Δ* mutant compared amongst themselves in each treatment; (**F**) WT compared to Sc*sod2Δ* mutant in each treatment. ns = not significant; * *p* < 0.04; *** *p* < 0.001.

Exposure of the four transformants to different mutagens allowed checking for phenotype complementation (Figure 4). The presence of Mp*SOD2* had no significant influence on the WT sensitivity phenotype (SM02) except when exposing the transformant to Paraquat (PAQ). Although the Sc*sod2Δ* mutant is only slightly sensitive to H_2_O_2_ when compared to the WT (SM03 *vs.* SM01) the change of mutagen-sensitivity phenotype of pLBF01-transformed Sc*sod2Δ* mutant (SM04) was always highly significant, except for UVC (Figure 4D, two-way analyses of variance—ANOVA). The Mp*SOD2-*containing WT transformant had a significantly altered phenotype after exposure to PAQ and DEN (Figure 4B). WT and *sod2Δ* mutant transformed with empty pRS313 (SM01 and SM03) had the same phenotypes as non-transformed cells to all tested mutagens.

Mp*SOD2* containing yeast transformants SM02 and SM04 exhibited higher resistance to H_2_O_2_ and PAQ when compared to the non Mp*SOD2*-containing strains SM01 and SM03. When exposed to H_2_O_2_ or PAQ non-Mp*SOD2-*containing yeast transformants, SM01 and SM03 were as resistant as the non-transformed WT (Figure 4A). A similar response of H_2_O_2_ sensitivity is known for *S. pombe* where a *sod2* deletion mutant also exhibits no clear H_2_O_2_ sensitivity phenotype [61]. However, when the WT and *sod2Δ* mutant were transformed with Mp*SOD2* (SM02 and SM04) they were slightly more resistant to H_2_O_2_ or PAQ than transformants harboring the empty vector (Figure 4A,B,E). Although yeast Sod2p is not directly involved in detoxification of H_2_O_2_ [20] introduction of a single copy of the Mp*SOD2* in the yeast mutant *sod2Δ* led to a significantly higher H_2_O_2_ and PAQ resistance as compared to that of the WT (Figure 4F).

This could be due to alterations in expression of ROS and to the level of anti-oxidant enzymes induced by an extra copy of Mp*SOD2*. Guo *et al.* [62] proposed that over-expression of MnSOD in mammalian cells results in redox alterations with subsequent expression of stress-responsive nuclear genes. In addition, over-expression of human MnSOD cDNA led to transient increases in mRNA levels of endogenous *sod2* and *txn2* genes in mouse NIH/3T3 cells and smaller increases in MnSOD and thioredoxin 2 protein content [63]. When constitutive gene expression led to over expression of MnSOD, the respective clones not only had higher MnSOD levels but often also showed alterations of other anti-oxidant enzyme profiles. These changes were considered adaptive phenotypes of MnSOD-over-expressing cells, which had to accommodate the elevated H_2_O_2_ production due to increased MnSOD activity [63,64]. Yan *et al.* [65] also observed increased expression of the endogenous *SOD2* gene in human lung fibroblasts following constitutive over-expression of exogenous *SOD2* cDNA. In addition, high-level expression of MnSOD from multi-copy plasmids rendered *S. pombe* *sod2* deletion mutant cells more resistant than the WT to superoxide-generating agents [61]. Therefore, the observed hyper-resistance phenotypes of yeast WT transformants induced by a single-copy Mp*SOD2* were expected.

Using the pro-mutagen DEN (that needs to be activated by redox cycling) we expected that either homologous or heterologous over expression of *SOD2* should induce a DEN sensitivity phenotype [66]. Nitrosamines are considered highly mutagenic and carcinogenic through *in vivo* generated β-oxidized metabolites, which lead mainly to α-carbon derivatives that are incorporated into DNA as a chain-shortened methyl group [67]. β-hydroxynitrosamines undergo a chemical retro-aldol-like, base-induced cleavage to methylalkynitrosamine [68]. Oxidizing enzymes play a possible role in the chain shortening, thus producing an alkoxy radical at the 2-carbon of the nitrosamine chain, which in turn fragments into aldehydes and ketones and shorter alkyl radicals. The production of the latter could be mediated by an appropriate metallo-enzyme [68,69]. Absence of MnSOD in yeast mutant *sod2Δ* (SM03) led to a DEN hyper-resistance phenotype as compared to the respective WT (SM01) (Figure 4C) whereas one copy of Mp*SOD2* (SM04) rendered the *sod2Δ* transformants WT-like (SM01) and two copies of *SOD2* genes in SM02, one from yeast, the other from *M. perniciosa*, conferred a DEN super-sensitivity phenotype (Figure 4C). Clearly, the presence of MnSOD, be it from yeast or from *M. perniciosa,* increased the DEN-induced cell damage which led to lower survival. In our Sc*sod2Δ* transformant SM04, this metallo-enzyme apparently is *Mp*Sod2p and the diminished survival in the WT transformant SM02 could be due to increased DNA alkylation by α-carboxy anions.

Both transformed and non-transformed yeast cells showed no significant phenotypic differences after UVC irradiation (Figure 4D), indicating that the small amount of ROS produced by this radiation [70] does not need Sod2p for removal.

**Figure 5:**
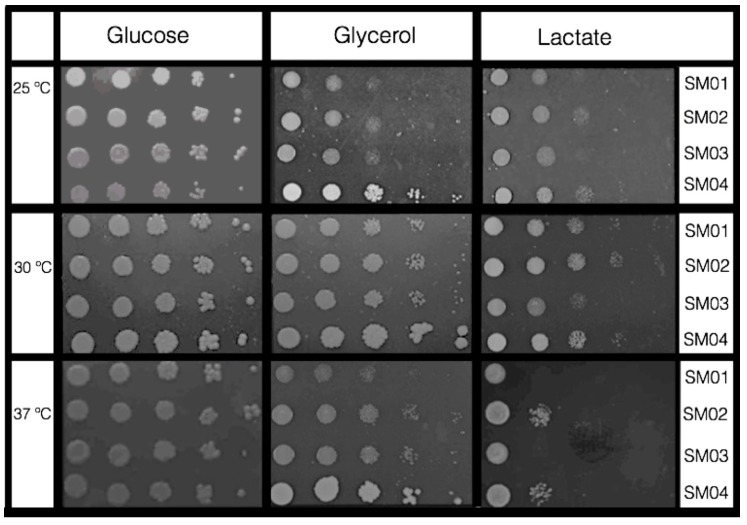
Temperature and carbon source-dependent growth of *S. cerevisiae* transformants harboring either plasmid pRS313 or pLBF01. Lines (fiveserial 1:10 dilutions): (1) SM01; (2) SM02; (3) SM03; (4) SM04.

Another typical phenotype of Sc*sod2*Δ is its sensitivity to lactate at different temperatures. Medium with glycerol or lactate shows that respiration on non-fermentable carbon sources generates superoxide radicals that inhibit the growth of cells lacking mitochondrial dismutase [71]. When we exposed the four yeast transformants to lactate, glucose and glycerol containing media at different growth temperatures (Figure 5), survival of the transformed yeast strains is influenced by the carbon source in the growth medium and the temperature.

Cell growth in 1% lactate was poor at all three incubation temperatures, especially at 37 °C. According to Krasowska *et al.* [71], lack of mitochondrial Sod2p in metabolism that relies on respiration of non-fermentable carbon sources generates growth-inhibiting superoxide radicals, especially in cells unable to cope with this oxidative stress. Clearly, the presence of functional MpSod2p in both SM02 and SM04 transformants enabled better growth and survival on lactate medium.

Cells grew well in glycerol (GLY) medium at 37 °C, especially the *MpSOD2-*containing SM02 and SM04 transformants (Figure 5). According to Whittaker [72] the mechanism of metal binding of MnSOD and FeSOD is strongly temperature-dependent: while incubation of the purified MnSOD apoprotein with metal salts at ambient temperatures did not restore SOD activity, re-activation could be achieved by heating the protein with Mn salts. MnSOD deficiency in *S. pombe* causes both osmotic and heat sensitivity, suggesting that in this organism MnSod2p plays a general role in protection against multiple stresses [61]. We may thus speculate that MpSod2p may have a general role in protection against heat sensitivity as well (Figure 4 and Figure 5; [22,28]), since functional over-expression of *Z-SOD2* genes (in multi-copy plasmid) of *Z. rouxii* improved salt resistance of some *S. cerevisiae* WT under acidic conditions [73].

Survival strategies of fungal plant pathogens in hosts are conserved and ROS-protection mechanisms may be one of the primary lines of fungal defense, guaranteeing a successful infection [74]. SOD enzymes are amongst the most important antioxidant metallo-enzymes protecting cells against oxidative stress arising from ROS produced by aerobic metabolism [9,20,52,72]. In *M. perniciosa*, a hemibiotrofic phytopathogen of *T. cacao*, general acquired resistance of dikaryotic cells pre-grown in GLY or shifted from glucose (GLU) to glycerol (GLY) media to the same set of mutagens (except DEN) has been reported [35]. This phenomenon could be explained by a pre-conditioned cellular response to oxidative stress generated by the non-fermentable carbon source GLY [43] and up-regulated expression of Mp*SOD2* may have a biological role in resistance of this fungus during infection of *T. cacao*.

The phenotypic changes introduced by heterologous expression of MpSod2p in the *sod2Δ* yeast mutant are a strong indication that the Mp*SOD2* gene has the same biological function in *M. perniciosa*. Further biochemical analysis is however necessary to prove conserved enzyme function.

**Figure 6:**
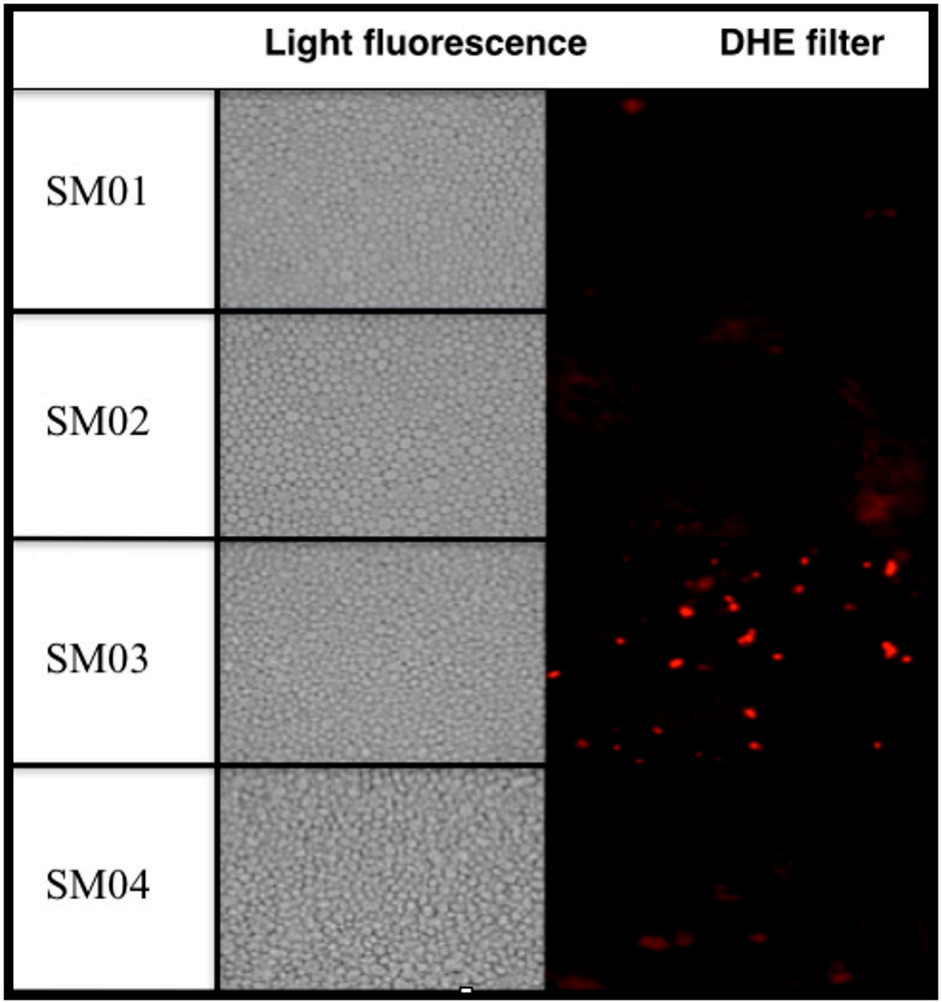
Visualization of oxidative stress via fluorescence microscopy of dihydroethidium (DHE)-stained strains.

As expected transformants SM01, SM02 and SM04 did not demonstrate physiological alteration in the presence of the pro-mutagen dihydroethidium (DHE), which indicates that there is low oxidative stress since the experiment was performed at basal level. However, SM03 presented strong coloration demonstrating a high level of oxidative stress (Figure 6).

## 3. Experimental Section

### 3.1. Strains and Growth Conditions

Yeast and bacterial strains, as well as plasmids used in this study are listed in Table 1. Stock cultures were usually grown in YPD media (2% glucose, 2% peptone, 1% yeast extract; 2% agar added for solid medium) at 30 °C, 180 rpm, in a gyratory water-bath shaker (New Brunswick Scientific^®^, Enfield, CT, USA) for 2 days to stationary phase of growth (STAT) with a cell titer of approximately 2 × 10^8^/mL. Selective growth of transformants was in SynCo medium (0.16% yeast nitrogen base (United States Biological, Swampscott, MA, USA), 2% glucose, 0.5% ammonium sulfate) supplemented with the appropriate essential amino acids and bases (40 μg/mL). To ascertain respiratory competence and for elimination of accumulated petites, all strains were pre-grown on YPG media (1% yeast extract, 2% peptone, 2% glycerol) before being grown in liquid YPD (1% yeast extract, 2% peptone, 2% glucose). Media, solutions and buffers were prepared according to [75].

**Table 1:** Bacterial and yeast strains used in this study.

Strains	Genotype	Source
DH5α	F-φ80lacZΔM15 Δ(lacZYA-argF)U169 deoR recA1 endA1 hsdR17(rk−, mk+) phoA supE44 thi-1 gyrA96 relA1 λ	Invitrogen
BY4742 (WT)	*MAT*α *his3*Δ1 *leu2*Δ0 *lys2*Δ0 *ura3*Δ0	EUROSCARF
BY4741 ( *sod2*Δ)	*MAT*α *his3*Δ1 *leu2*Δ0 *met15*Δ0 *ura3*Δ0 *SOD2*: KanMX4	EUROSCARF
SM01	Same as BY4742 containing pRS313	This study
SM02	Same as BY4742 containing Mp*SD2*	This study
SM03	Same as BY4741 containing pRS313	This study
SM04	Same as BY4741 containing Mp*SOD2*	This study
Plasmid Name	Relevant Sequence Identification	Source
pDNR-Lib	CLONTECH containing Mp*SOD2*	Acassia BL Pires
pRS313	Single copy plasmid, *HIS1*protrotrophy	[76]
pLBF01	pRS313 Mp*SOD2*	This work

### 3.2. Identification of an MpSOD2 cDNA Clone and Sequence Analysis

Mp*SOD2* cDNA was identified from a previously constructed mycelial cDNA library in the pDNR-LIB plasmid using DB SMART Creator cDNA (Creator SMART cDNA Library Construction Kit, Clontech, Palo Alto, CA, USA) that had been derived from primordia and mature basidiomata [77] (plate identity: CP02-EC-001-002-C06-UE.F, clone C06). Based on its similarity to known *SOD*2 genes an open reading frame (ORF) analysis was performed (ORFinder program, Lasergene, Madison, WI, USA) followed by homology search with BLAST [78] against sequences in the NCBI database. ClustalW was used for multiple sequence alignment of amino acid sequence [79].

Phylogenetic analysis was by Neighbor-Joining method [58] using program MEGA4 [80]. The bootstrap consensus tree was inferred from 1000 replicates [81]. Evolutionary distances were estimated using the Poisson correction method [82]. We eliminated all positions containing gaps and missing data from the analysis (complete deletion option). There were 93 positions in the final dataset. Phylogenetic analyses were conducted in MEGA4. The sequence CDS was predicted by Augustus Gene Predictor v2.7 (Department of Bioinformatics, University of Göttingen, Göttingen, Alemanha) [83].

### 3.3. Amplification of MpSOD2 and Sub-Cloning Plasmid pRS313 (Yeast Centromere Vector with a HIS3 Marker and a Multiple Cloning Site)

Bacterial clone C06 containing Mp*SOD2* was grown overnight in LBC medium (NaCl 1%, tryptone 1%, yeast extract 0.5%; 34 μg/L chloramphenicol) and plasmid DNA was extracted via alkaline lysis [41]. The entire putative Mp*SOD2* gene was amplified with specific primers of pDNR-LIB (Primer sequence M13F: 5ʹ-CGCCAGGGTTTTCCCAGTCACGAC-3ʹ, M13R: 5ʹ-TCACACAGGAAACAGCTATGAC-3ʹ). DNA was amplified in a thermo cycler (Mastercycler Eppendorf^®^, Hamburg, Germany) at 94 °C for 5 min (1 cycle); 94 °C for 1 min, 58 °C for 1 min and 72 °C for 1 min (30 cycles) and 72 °C for 7 min (1 cycle).

Amplification products were run on 1% Tris-borate-EDTA (TBE)-agarose gel followed by fragment purification with the QIAquick Gel Extraction Kit, Qiagen (Venlo, Limburg, The Netherlands). Both amplified fragments of Mp*SOD2* and shuttle vector pRS313 were digested with restriction enzyme *Sma*I (Fermentas International, Vilnius, Lithuania, for 16 h at 30 °C), followed by a treatment for 4–12 h at 11 °C with Klenow enzyme (Applied Biosystems^®^, Life Technologies, Waltham, MA, USA) and incubated for 1 h at 16 °C with T4 DNA Ligase (Fermentas International) for blunt-end ligation. The entire reaction mixture was incubated with competent DH5α cells (Invitrogen™, Life Technologies, Waltham, MA, USA) and transformants were selected by blue/white screening. Putative clones were individually grown on LBA solid media and confirmed by a PCR run of each colony. Then each candidate was grown in LBA medium (NaCl 1%, tryptone 1%, yeast extract 0.5%; 50 μg/L ampicillin) for 13–18 h at 37 °C at 200 rpm agitation. DNA was extracted from each putative clone [84] and Mp*SOD2-*containing clones confirmed via PCR (internal primers of sequence Mp*SOD*2F: 5ʹ-TGCTCTCGAGCCCTACATTT-3ʹ, Mp*SOD*2R: 5ʹ-AACGCTTAGTGTCCGGTTTG-3ʹ; conditions of PCR as described above). The confirmed construct of plasmid pRS313 (Mp*SOD2*) was named pLBF01.

### 3.4. Cloning of pRS313 (MpSOD2) in S. cerevisiae Yeast Mutant

Yeast mutant Sc*sod2Δ* and its isogenic WT were transformed with pLBF01 by the LiAc/SS-DNA/PEG method [85] and putative transformants were selected in SynCo medium lacking histidine (SC-His). Selected transformants were tested for their sensitivity to paraquat, H_2_O_2_, diethylnitrosamine, and UVC (conditions below).

### 3.5. Mutagen Exposure and Cell Survival

Sensitivity of *S. cerevisiae/MpSOD2* transformants to different mutagens was determined as described by [86] on SC-His media supplemented (at the time of pouring the liquid agar media) with the following oxidative stress-inducing chemicals: paraquat (PAQ, 100 to 400 μM), hydrogen peroxide (H_2_O_2_, 1 to 3 mM), diethylnitrosamine (DEN, 100 to 160 mM). We determined sensitivity to UVC radiation by irradiation of agar-plated tranformant cells with exposure doses between 0 and 150 J/m^2^ (Spectrolinker, Spectronics Corp., Westburg, NY, USA). Results are expressed as the percentage of survival related to the untreated controls and are the mean of at least three independent experiments. The error bars represent standard deviation as calculated by the GraphPad Prism^®^ program (GraphPad Software Inc., San Diego, CA, USA). Yeast transformants drop tests show cell growth in each 5 μL drops of a serial 1:10 dilution (10^7^ to 10^3^ cells/mL) placed on SC-His media containing one of the following carbon sources: glucose, glycerol or lactate (2%). Incubation was for 5 days at 3 different temperatures, 25, 30 and 37 °C. Photos represent one of at least three triplicates.

### 3.6. Fluorescence Assay

A stock solution (1 mg/mL) of the fluorogenic probe dihydroethidium (DHE, Sigma Aldrich, St. Louis, MO, USA) was prepared by dissolving it in dimethyl sulfoxide (Sigma Aldrich, St. Louis, MO, USA). One mL of cells in LOG phase was stained by addition of 1 μL of stock solution and mixed by inversion, then incubated for 30 min 35 °C, washed 3 times with saline, and finally resuspended in 100 μL. An aliquot was used to check oxidative/reductive stress of the cells. Cytosolic DHE when oxidized by ROS (singlet oxygen, hydroxyl radicals, superoxide, hydroperoxides and peroxides) yields ethidium, which intercalates with a cell’s DNA and fluoresces bright red (605 nm) [87] when observed under fluorescence microscope DMRA2 (Leica, Wetzlar, Germany) attached with DHE filter. Images were captured using a 40× objective under bright field as well as under fluorescent filters using the IM50 software (Leica). Photos represent cells from at least three independent experiments.

## 4. Conclusions

In this work, the putative Mp*SOD2* gene was cloned using standard molecular biology tools and the phenotypical complementation of the yeast mutant *sod2Δ* was demonstrated via classical genetics. The clone containing Mp*SOD2* gene, named SM04, showed increased resistance phenotypes against oxidative stress-inducing H_2_O_2_ and paraquat, and enhanced growth at 37 °C on the non-fermentable carbon source lactate. Surprisingly, the presence of the *SOD2* gene either from yeast or from *M. perniciosa*, rendered higher sensitivity of the cells against treatment with the pro-mutagen diethylnitrosamine (DEN) that requires oxidation to become an active mutagen/carcinogen. Absence of Mp*SOD2* in the yeast *sod2Δ* mutant led to DEN hyper-resistance while introduction of a single copy of this gene restored the yeast wild-type phenotype. Fluorescence assay using DHE was performed to observe basal levels of yeast cell oxidative stress and results demonstrated that the *sod2Δ* mutant has a much higher level of intrinsic oxidative stress, which could be abolished by introducing Mp*SOD2*. Taken together, this data indicates that MpSod2p is functionally expressed in yeast *S. cerevisiae*. This is the first report of *in vivo* functional expression of a *M. perniciosa* gene.
